# Pulmonary Arterial Hypertension in Adult-Onset Still's Disease: Rapid Response to Anakinra

**DOI:** 10.1155/2012/537613

**Published:** 2012-08-29

**Authors:** Marc Campos, Elena Schiopu

**Affiliations:** Division of Rheumatology, University of Michigan, P.O. Box 481, Ann Arbor, MI 48109, USA

## Abstract

Adult-onset Still's disease (AOSD) is a rare inflammatory condition characterized by spiking quotidian fever, rash, chronic arthralgia, leukocytosis, and occasional pulmonary involvement such as pleural effusion and transient pulmonary infiltrates. Pulmonary arterial hypertension (PAH) is a rare pulmonary complication of AOSD, and we are aware of only 5 cases reported in the literature. We report the case of a 27-year-old woman of Middle Eastern descent, with a 7-year history of AOSD, who developed severe pulmonary arterial hypertension (PAH). After unsuccessful exposure to various immunosuppressive regimens, shortly following the initiation of anakinra, an interleukin-1 (IL-1) receptor antagonist, her disease became quiescent and the PAH resolved. With this case report, we hope to show that anakinra, either by virtue of controlling the overall inflammation in AOSD, or by direct effect on the pulmonary microangiopathy, can improve severe PAH.

## 1. Introduction

Adult-onset Still's disease (AOSD) is a systemic inflammatory disorder characterized by a variety of clinical features, including intermittent fever, arthritis, evanescent rash, neutrophilic leukocytosis, polyserositis, lymphadenopathy, splenomegaly, and other systemic symptoms [1]. The etiology of AOSD is unknown although a paradigm of genetic predisposition combined with viral triggers has been proposed. The annual incidence has been estimated by a French study to be 0.16 cases per 100,000 persons, with equal representation of the genders [2]. According to the same study, there is a bimodal age distribution, with a first peak at 15–25 years of age, and 36–45 years. Due to the heterogeneity of the presenting symptoms, multiple classification criteria were proposed, but the highest sensitivity for a definite diagnosis of AOSD is claimed by the Yamaguchi criteria [3]. The Yamaguchi criteria require the presence of five features, with at least two being major criteria: there are four major criteria (fever, arthralgias/arthritis, specific rash, and leukocytosis) and a few minor criteria (sore throat, lymphadenopathy, hepato- and splenomegaly, abnormal liver function tests, negative tests for antinuclear antibody, and rheumatoid factor). Although nonspecific, markedly elevated ferritin levels have been described to help in diagnosis and monitoring in patients with AOSD [4]. Cardiopulmonary disease has been described in 30–40% of the patients with AOSD. Serositis, mild cough, pleuritic chest pain, and dyspnea are the symptoms of lung involvement, while the pathology encompasses acute pneumonitis manifested as transient infiltrates or severe interstitial lung disease [5]. The treatment of AOSD is greatly directed at the inflammatory component. The traditional approach, depending on the severity of the disease, recommended glucocorticoids initiated at 0.5–1 mg/kg/day, followed quickly by a disease-modifying antirheumatic drug (DMARD), such as azathioprine, methotrexate, or leflunomide. The biologic agents have since become the main avenue in patients with steroid-resistant disease, although approximately 70% of the patients respond to steroids alone [6]. Anakinra, a recombinant human IL-1 receptor antagonist, has been shown to rapidly reduce the systemic inflammatory reactions in small series of patients with refractory AOSD [7]. Further, a case series of 25 patients showed rapid resolution of AOSD clinical activity within 6 days and the response was maintained at one year [8]. Newer approaches, including tocilizumab, an anti-IL-6 receptor monoclonal antibody [9] are being proposed.

We report a case of AOSD refractory to glucocorticoids and DMARD combination of azathioprine and hydroxy-chloroquine, complicated by severe pulmonary arterial hypertension, with rapid response to anakinra, and normalization of the pulmonary pressures. We will review the existing literature and discuss the response to treatment.

## 2. Case Report

A 27-year-old female of Middle Eastern descent with a history of AOSD for 7 years based on persistent quotidian fevers, evanescent rash, polyarthritis, lymphadenopathy, splenomegaly, leukocytosis, and polyclonal hypergammaglobulinemia was evaluated in our institution for acute onset of severe dyspnea, requiring oxygen supplementation, shortly after a successful pregnancy and uncomplicated delivery. Her rheumatological care for AOSD was conducted in the United Arab Emirates and it included prednisone (unspecified doses), azathioprine, and chloroquine. The patient was initially found to be tachycardic and hypoxic, with a significant cardiac murmur, which prompted an echocardiographic evaluation that revealed the following findings: estimated right ventricular systolic pressure (RVSP) of 100 mmHg, severe tricuspid regurgitation, dilated right atrium, and ventricle and moderate amount of pulmonic valvular regurgitation. Based on these findings, she was referred to the pulmonary hypertension clinic at our institution and hospitalized for further work up.

Her physical examination revealed a cushingoid female in moderate respiratory distress, afebrile, with a pulse of 97 beats/min, a respiratory rate of 20/min, and a blood pressure of 106/78 mmHg, saturating 97% on room air. The lung fields were clear to auscultation, and cardiac examination revealed: right ventricular heave, a loud pulmonic component, and a palpable pulmonary artery, along with a varying murmur of tricuspid regurgitation. The jugular venous distension was up to her earlobes at 45 degrees. There was trace peripheral edema, no evidence of synovitis or rash, and absent hepato- or splenomegaly.

At this time, her medication included prednisone 15 mg/day and azathioprine 50 mg twice daily. The laboratory investigation showed normal C-reactive protein and sedimentation rate, negative hepatitis B and C serology, negative rheumatoid factor and ANA, normal ferritin, complements (C3 and C4) levels, slightly elevated AST and ALT, and normal complete blood count. The chest radiograph showed right atrial and ventricular dilatation and small right pleural effusion. The electrocardiogram showed signs of right axis deviation, right ventricular hypertrophy, and right atrial enlargement, but normal sinus rhythm. She underwent a CT angiography that showed no evidence of pulmonary embolism or interstitial lung disease. The examining cardiologist classified her, based on current symptoms, as New York Heart Association Class IIIB. Based on these preliminary studies, the patient underwent a right and left heart catheterization. Her left heart catheterization revealed completely normal coronary arteries. Presence of pulmonary hypertension was confirmed, based on main pulmonary artery pressure (PAP) of 73/35 mmHg (mean 48 mmHg), pulmonary capillary wedge pressure (PCWP) of 10 mmHg, right atrium pressure of 9 mmHg, cardiac output (CO) by thermodilution was 4.1 L/min, and the calculated pulmonary vascular resistance was 9.3 Wood Units. 

**Figure 1:**
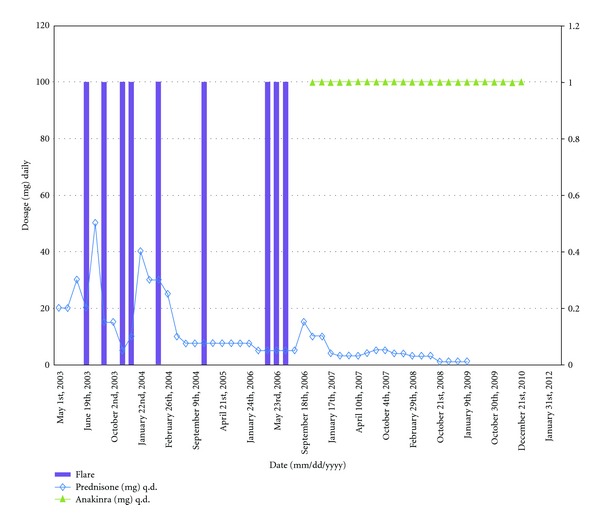
Improvement in flares and the decline of the prednisone dose after initiation of anakinra.

**Figure 2:**
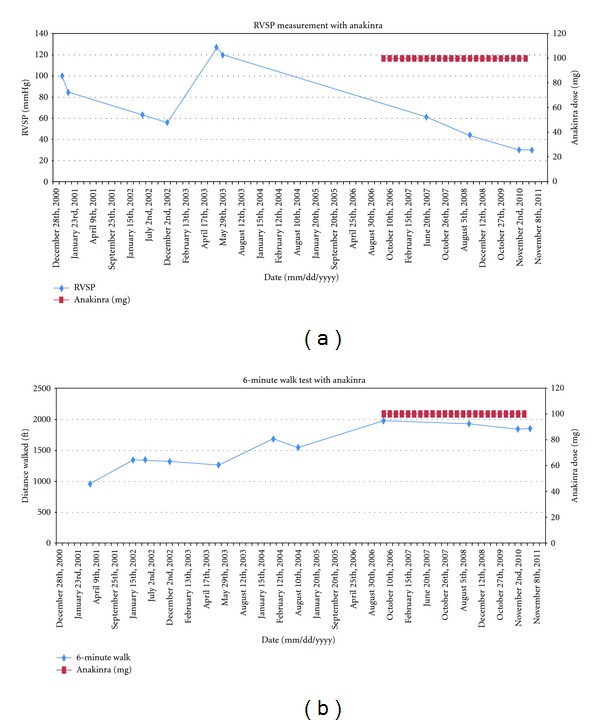
(a) Improvement in 6MWT after anakinra initiation; (b) improvement in RVSP after anakinra initiation. 6MWT: six minute walk test; RVSP : right ventricular systolic pressure.

The patient was started on amlodipine at 10 mg daily and the rest of immunomodulatory drugs were continued, along with careful monthly clinical follow up. The patient continued to remain dyspneic and she continued to have flares characterized by fevers, rash, and uncontrolled arthralgias (Figure 1). She had moderate improvement of her RVSP by echocardiogram, but her functional class and dyspnea continue to worsen with the flares (Figure 2). Her pulmonary artery pressure continued to stay in the moderate range, prompting increase of the dose of amlodipine to 20 mg daily. A right heart catheterization (RHC) one year following the first one showed PAP of 48/19 mmHg (mean not reported), PCWP of 10 mmHg, and CO of 5.2 L/min. Within another year, her RHC measurements were: PAP of 50/22 (mean of 34 mmHg), PCWP of 9 mmHg, and CO of 6.7 L/min. Despite an overall sense of improvement, the patient continued to have clinical flares and dyspnea. Approximately 5 years from the diagnosis of PAH, following a rocky clinical course, the patient was initiated on anakinra, 100 mg daily, by subcutaneous injections. The patient experienced rapid resolution of her systemic symptoms, and due to the lack of recurrence of her flares, the prednisone was weaned down and stopped within 18 months (Figure 1). She also noticed improvement in her effort tolerance which is evidenced in her performance on six-minute walk tests (Figure 2(a)). She continues to be followed up in the rheumatology clinic on quarterly basis but she is seen only yearly in the pulmonary hypertension clinic.

## 3. Discussion

We have described a patient with AOSD, refractory to glucocorticoids, who developed severe PAH seven years after the onset of her disease. The PAH seemed to respond to the calcium channel blocker amlodipine, but it was completely resolved upon initiation of IL-1 blockade with anakinra, 5 years later. The patient met the American College of Chest Physicians (ACCP) criteria for prepulmonary hypertension [10]. Based on history, there was no history of sleep disorder, chronic liver disease, HIV infection, anorexigen ingestion, or congenital heart disease. More so, other causes of PAH (such as left heart disease, interstitial lung disease, and chronic thromboembolic lung disease) were excluded by the echocardiogram and the CT angiogram. We feel confident that she had WHO group I PAH. 

We performed a literature search to identify described cases of group I PAH in the setting of AOSD. We identified a total of 5 cases described between 1990 and 2011. The first reported case was in 1990, of a 29-year-old woman who developed severe, progressive, precapillary PAH 2.5 years after her diagnosis [11]. A retrospective analysis of 19 patients with PAH and various autoimmune diseases was done in northern Taiwan, where PAH has been reported seldom, and revealed that 2 patients had AOSD; in this study, 5 patients died and the RVSP correlated with the levels of serum uric acid (*r* = 0.686, *P* = 0.001) [12]. Another case report described a 29-year-old woman, who 9 years after a diagnosis of AOSD, developed PAH (mean PAP 33 mmHg) responsive to vasodilator infusion, but died 2.5 months later of right heart failure, despite initiation of anakinra [13]. Lastly, a case report from Hyderabad, India, described an 18-year-old female with new onset AOSD who developed severe PAH (mean PAP of 65 mmHg); in the absence of other causes, the authors concluded that the PAH was secondary to the AOSD-related pulmonary microangiopathy [14].

We now report a case of PAH in AOSD which responded rapidly and dramatically to anakinra. Although anakinra was reported to offer rapid control of the systemic manifestations of AOSD [7], there has been no report of improvement of the pulmonary pressures and functional status in a patient with AOSD-PAH. As it is often the case with rare conditions characterized by high symptomatic variability, it is difficult to organize our thoughts when it comes to clinical patterns of causality. It is interesting that our patient, who was diagnosed in 2001, was not exposed to PAH therapy, including the “modern” era (described as starting with the first oral PAH medication in 2002) [15]. Despite availability of PAH-specific therapy, the mortality associated with PAH remains significant [15]. Early diagnosis, classification, and initiation of a disease specific treatment algorithm might improve survival.

Our patient had refractory AOSD, characterized by persistence of flares and elevated inflammatory markers, and in that setting developed PAH. The PAH responded, but not completely, to amlodipine, although her AOSD remained particularly inflammatory. The initiation of IL-1 receptor blockade curtailed both the AOSD and the PAH. It is possible that this was part of the natural history of the disease, as it is described in patients with an intermittent pattern (flares with complete remission between flares and decreasing severity of the flares with time) [16]. However, we believe that the inflammatory burden present in AOSD is most likely the driver for the pulmonary microangiopathy, much like the renal involvement and thrombotic thrombocytopenic purpura in AOSD. It is likely that screening for presence of PAH in patients with AOSD will yield a higher prevalence of AOSD-PAH and will help prevent progressive disease and associated mortality. There is evidence that anakinra lowers pulmonary artery pressure in neonatal surfactant depleted piglet model and further reduces the early proinflammatory pulmonary reaction [17].

The pathophysiology of WHO Group I PAH, specifically PAH associated with autoimmune disorders is unknown. In general, PAH is characterized by proliferation of endothelial cells and expansion of the vascular smooth muscle and adventitial cells, leading to obliteration of the lumen, particularly in the precapillary arterioles [18]. With the increased knowledge about PAH and the ability to recognize its association with autoimmune condition, it is now clearer than ever that autoimmunity plays a significant role in development of PAH. The discussion is starting around regulatory T-cells playing a role in preventing B-cell activity, and potentially leading to the severe angioproliferative pulmonary hypertension that seems to characterize various connective tissue disorders (CTDs). There is a lack of systematic evaluation of how immunosuppressive regimens will affect pulmonary artery pressures in CTD-PAH. It is possible that in CTD-PAH the endothelial cells are apoptosis-resistant, similar to the malignant cells, therefore, much more resistant to traditional PAH therapies [18]. Based on our case, it is likely that immunosuppressives, such as biological agents, alone or in addition to PAH-specific medications, might improve outcomes in AOSD-PAH.

To conclude, we report the first case of anakinra-responsive PAH in the setting of AOSD. We think that PAH should be added as part of the pulmonary manifestations of AOSD, and that clinicians should consider screening for this complication. AOSD should also be added to the WHO Group I PAH secondary to CTDs, and the treatment of AOSD-PAH should be customized to target the inflammatory component of the disease.
